# Laparoscopic Presacral Neurectomy in the Treatment of Primary Dysmenorrhea

**DOI:** 10.1155/DTE.1.223

**Published:** 1995

**Authors:** K. K. Chu, E. P. Chen, S. D. Chang, Y. K. Soong

**Affiliations:** 1 Department of Obstetrics/Gynecology Chang Gung Memorial Hospital Keelung Taiwan China; 2 222, Mai-Chin Road Keelung Taiwan China

## Abstract

Sixty-one laparoscopic presacral neurectomies were performed in Chang Gung Memorial Hospital, Keelung Center over a 1-year period for patients with primary dysmenorrhea who failed to respond to medical management. Eighty-three percent (50) had complete relief of pain, 10% (6) had significant relief, 3.5% (2) had moderate relief, and 3.5% (2) had no relief from their dysmenorrhea at their first menstruation after the procedure. One vascular complication requiring laparotomy occurred. Average total operation time was 24 minutes, and the average hospital stay was 2 days. Six months follow-up was done in 35 patients with complete symptom relief, and all reported continued relief of menstrual pain. The efficacy of laparoscopic presacral neurectomy for the pain relief of primary dysmenorrhea is justified in this report.


*Diagnostic and Therapeutic Endoscopy*, 1995, Vol. 1, pp. 223–225
Reprints available directly from the publisher
Photocopying permitted by license only

© 1995 Harwood Academic Publishers GmbH
Printed in Singapore

### Laparoscopic Presacral Neurectomy in the Treatment of Primary Dysmenorrhea

K.K. CHU, F.P. CHEN, S.D. CHANG, and Y.K. SOONG

*Department of Obstetrics/Gynecology, Chang Gung Memorial Hospital, Keelung, Taiwan, R.O.C.*

*(Received May 25, 1994; in final form December 12, 1994)*

Sixty-one laparoscopic presacral neurectomies were performed in Chang Gung Memorial Hospital, Keelung Center over a 1-year period for patients with primary dysmenorrhea who failed to respond to medical management. Eighty-three percent (50) had complete relief of pain, 10% (6) had significant relief, 3.5% (2) had moderate relief, and 3.5% (2) had no relief from their dysmenorrhea at their first menstruation after the procedure. One vascular complication requiring laparotomy occurred. Average total operation time was 24 minutes, and the average hospital stay was 2 days. Six months follow-up was done in 35 patients with complete symptom relief, and all reported continued relief of menstrual pain. The efficacy of laparoscopic presacral neurectomy for the pain relief of primary dysmenorrhea is justified in this report.

**KEY WORDS:** laparoscopy, presacral neurectomy, dysmenorrhea

### INTRODUCTION

Before 1960, the approach for treatment of dysmenorrheic patients was often presacral neurectomy performed via laparotomy (1). Since then, the management of dysmenorrhea has been replaced largely by medical management with nonsteroidal antiinflammatory drugs, oral contraceptives, danazol, and gonadotropin-releasing hormone analogs. However, more than 25% of dysmenorrheic patients fail to respond to medical treatment (2). Conservative alternative treatment modalities (3) have been sought in an attempt to manage medical therapy failures. A laparoscopic approach to the classic presacral neurectomy was devised and implemented (4). This article reports on the recent experience of laparoscopic presacral neurectomy at Chang Gung Memorial Hospital, Keelung Center, when used in those patients who failed medical therapy for primary dysmenorrhea.

### MATERIALS AND METHODS

During the 1-year period between November 1992 and October 1993, 61 patients underwent operative laparoscopy for dysmenorrhea, and when no definite pelvic pathology was revealed, laparoscopic presacral neurectomy was undertaken. These patients were questioned very carefully about the medication they took for pain relief and a thorough history taken and physical examination performed. Patients with dysmenorrhea were included only if nonsteroidal analgesics were ineffective for over 6 months and laparoscopy revealed a normal pelvis.

At the time of surgery, laparoscopy was performed using general anesthesia with endotracheal intubation. Pneumoperitoneum was established as usual. A three-puncture technique was used with one 10-mm subumbilical endoscope port and two 5-mm ports on each side midway between the umbilicus and the iliac crest. These two ports are for instruments insertion such as grasping forceps, bipolar forceps, or endoshears (scissors with unipolar cautery).

After thorough examination of the pelvis by laparoscopy, the great vessels of the aortic bifurcation and bilateral common iliac arteries were identified; the ureter was identified on the right side, and the inferior mesentery artery and superior hemorrhoidal vessel were identified on the left. The peritoneum overlying the sacropromontary 1 cm caudal to the aortic bifurcation was elevated and incised; the underlying adipose tissue was grasped and dissected bluntly, cauterized, and cut; and the subperitoneum adipose tissue was removed first. By successive cauterization and cutting, nerve plexuses then

Address for correspondence: Dr. Chu Kiu-Kwong, 222, Mai-Chin Road, Keelung, Taiwan, R.O.C., Department of Obstetrics/Gynecology, Chang Gung Memorial Hospital, Keelung Center.

were identified and freed from their underlying tissue containing the left common iliac vein and middle sacral vein. Once the tissue overlying the whole promontary was excised, the retroperitoneal space was irrigated and left open for spontaneous healing. Care should be taken to avoid injury of the presacral vessels (Fig. 1).

Postoperatively, patients were kept in the hospital or allowed to leave according to anesthesia conditions. Patients were questioned about pain relief at their first menstrual cycle after the procedure. Those who were totally freed from preoperation analgesic medication were considered to have achieved complete relief. Significant-relief patients were those who required one third or less of the minimal preoperative analgesics; moderate-relief patients were those who required half the preoperative dosage; and no-relief patients required more than half the dosage of preoperation analgesics.

### RESULTS

Sixty-one patients with primary dysmenorrhea underwent laparoscopic presacral neurectomy without complications except one who required immediate laparotomy to control bleeding from the great vessels. This patient was excluded from the statistics. At the visit after the first menstrual cycle postoperatively, of 60 patients, 83% (50) had complete relief, 10% (6) had significant relief, 3.5% (2) had moderate relief, and 3.5% (2) had no relief from their dysmenorrhea. All were back to full activities by 10 days after the operation. Thirty-five patients with complete symptom relief have been followed-up for at least 6 months, and all reported continued relief of the menstrual pain. The mean operation time for laparoscopic presacral neurectomy was 24 minutes in our series, and the average hospital stay was 2 days.

### DISCUSSION

Antiprostaglandins and oral contraceptives are very effective in treating patients with dysmenorrhea (5). Occasionally, medical therapy is ineffective (6), and conservative surgical approach should be recommended.

Presacral neurectomy has proved its efficacy and success in controlling dysmenorrhea and has been reported infrequently in recent years (6,7). In a randomized, controlled trial, presacral neurectomy was found to be effective in 88% of study patients, compared with 0% in nonneurectomy controls (8). A controlled trial in quoted.

Since there has been such an emphasis on outpatient treatment and minimal surgery, a laparoscopic approach to the classic presacral neurectomy was devised and implemented (4).

In our article, over 90% of the primary dysmenorrhea patients gained significent pain relief from dysmenorrhea, and all 35 patients with 6 months (1) follow-up showed continued effectiveness. No serious complication was noted except in one patient with massive bleeding from the left common iliac vein. The complication can be prevented. The nerve plexus overlying the vein should be promptly identified, and blunt dissection to separate the tissue from the great vessel is important to prevent bleeding. The use

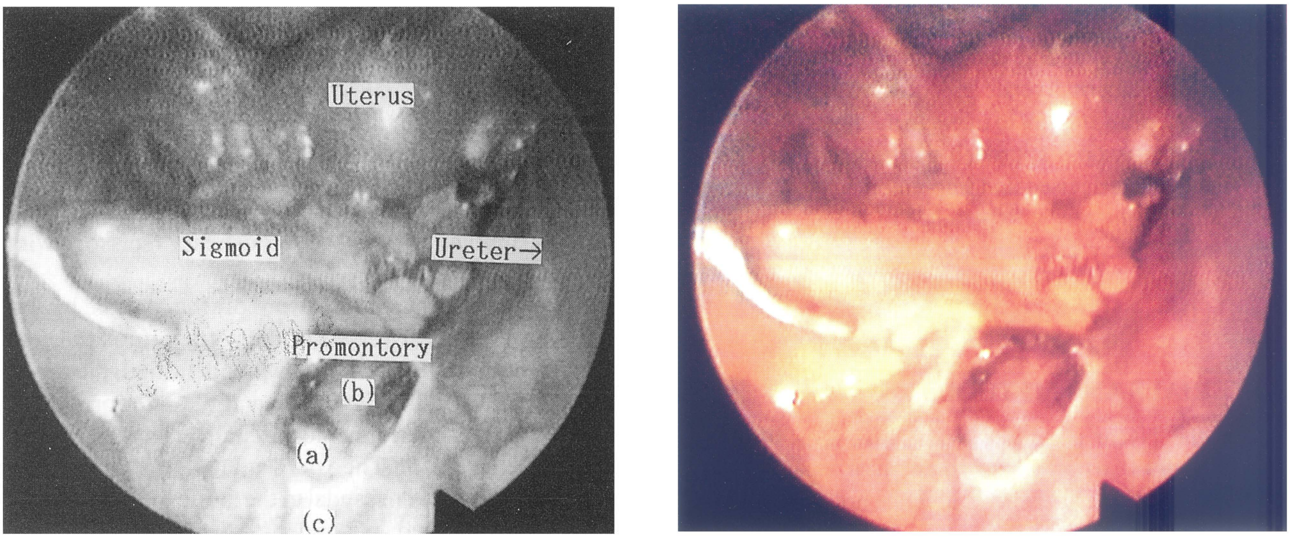

**Figure 1** The superior hypogastric plexus was completely removed from the underlying left common iliac vein (*a*) and middle sacral vein (*b*), located 1 cm caudal to the aortic bifurcation (*c*).

of an argon-beam coagulator also can separate and damage a vessel before one realizes that the vessel is in the way.

The operation and recovery times are brief. The procedure of laparoscopic presacral neurectomy could be easily accomplished by laparoscopist. It also could be advantageous as an adjuvant procedure for patients associated with pelvic pathologies such as endometriosis or pelvic inflammatory disease.

Laparoscopic presacral neurectomy seem to have all the epidemiologic earmarks of an effective procedure, which includes anatomic rationale and many observational studies with consistently reproducible results, and because it is a simple laparoscopic procedure, its application will increase and it will become another part of the surgical armamentarium for treating pelvic pain.

### SUMMARY

In cases of primary dysmenorrhea, significant relief of symptoms can be accomplished by the simple technique of laparoscopic presacral neurectomy. This can be performed as an outpatient procedure and proves to be an effective alternative treatment for dysmenorrhea failing to respond to medical treatment.

### REFERENCES

1. Weigs JV. Excision of the superior hypogastric plexus (presacral nerve) for primary dysmenorrhea. Surg Gynecol Obstet, 1939;68:723–732.
2. Dawood YM. Premenstrual Syndrome and Dysmenorrhea. Urban and Schwarzenberg, Baltimore. 1985.
3. Lichten EM, Bombard J.: Surgical treatment of primary dysmenorrhea with laparoscopic uterive nerve ablation. J Reprod Med 1987;32:37–41.
4. Perez JJ. Laparoscopic presacral neurectomy: results of the first 25 cases. J Reprod Med 1990;35:625–630.
5. Henzl MR. Dysmenorrhea: achievement and challenge. Sex Med Today 1985;9:8–18.
6. Lee RB, Sonte K, Magelssen D et al. Presacral neurectomy for chronic pelvic pain. Obstet Gynecol, 1986;68:517–521.
7. Blach WT. Use of presacral sympathetomy in the treatment of dysmenorrhea. Am J Obstet Gynecol 1964;89:16–22.
8. Tjaden B, Schlaff WD, Kimballa et al. The efficacy of presacral neurectomy for the relief of midline dysmenorrhea. Obstet Gynecol 1990;76:89–91.

